# Informal caregivers in Germany – who are they and which risks and resources do they have?

**DOI:** 10.3389/fpubh.2023.1058517

**Published:** 2023-02-16

**Authors:** Judith Fuchs, Beate Gaertner, Alexander Rommel, Anne Starker

**Affiliations:** Department of Epidemiology and Health Monitoring, Robert Koch Institute, Berlin, Germany

**Keywords:** informal care, population-based study, Health Monitoring, Germany, health status, behavioral risk factors, social risk factors

## Abstract

**Background:**

The aim of this study is to describe the social characteristics, the health and living situation and the prevalence of behavioral risk factors of adult informal caregivers compared to non-caregivers in Germany.

**Methods:**

We used data from the German Health Update (GEDA 2019/2020-EHIS survey) which is a cross-sectional population-based health interview survey conducted between 04/2019 and 09/2020. The sample comprised 22,646 adults living in private households. Three mutually exclusive groups of providing informal care or assistance were differentiated: intense caregivers (informal care ≥10 h/week), less-intense caregivers (informal care<10 h/week) and non-caregivers. For the three groups weighted prevalences of social characteristics, health status (self-perceived health, health-related activity limitations, chronic diseases, low back disorder or other chronic back defect, depressive symptoms), behavioral risk factors (at-risk drinking, current smoking, insufficient physical activity, non-daily fruit and vegetable consumption, obesity) and social risk factors (single household, low social support) were calculated and stratified by gender. Separate regression analyses adjusted for age-group were conducted to identify significant differences between intense and less-intense caregivers vs. non-caregivers, respectively.

**Results:**

Overall, 6.5% were intense caregivers, 15.2% less-intense caregivers and 78.3% non-caregivers. Women provided care more often (23.9%) than men (19.3%). Informal care was most frequently provided in the age group of 45 to 64 years. Intense caregivers reported worse health status, were more often current smokers, physical inactive, obese and lived less often alone than non-caregivers. However, in age-group adjusted regression analyses only few significant differences were seen: Female and male intense caregivers had more often a low back disorder and lived less often alone compared to non-caregivers. In addition, male intense care-givers reported more often worse self-perceived health, health-related activity limitation, and the presence of chronic diseases. In contrast, less-intense caregivers and non-caregivers differed in favor of the less-intense caregivers.

**Discussion:**

A substantial proportion of the adult German population provides informal care regularly, especially women. Intense caregivers are a vulnerable group for negative health outcomes, especially men. In particular measures to prevent low back disorder should be provided. As the necessity of providing informal care will probably increase in the future, this will be important for the society and public health.

## 1. Introduction

The progression of the demographic change and the increase in life expectancy are leading to a steady increase in the share of older people with physical and cognitive impairments frequently in need of long-term care.

A large part of this long-term care is provided by informal caregivers, usually family members. Since the introduction of the statutory long-term care insurance in Germany in 1995, the provision of informal care services can be supported by cash benefits or in-kind benefits if the Medical Service of the Health Insurance Funds certified a need of care. Currently, around 4.1 million people in Germany claim benefits from long-term care insurance every month. Most of the recipients receive outpatient care (about 3.3 million), 2.1 million are cared for at home by informal caregivers (mostly relatives), and around 818,000 people receive inpatient care.

In Germany, the criteria for needing long-term care and entitlement to long-term care benefits from the long-term care insurance are regulated by law. If an entitlement exists, people in need of care can decide how and by whom they will be cared for, with various forms or facilities available (outpatient care, nursing home, alternative forms of living). The choice depends on the severity of the need for care, but also on the circumstances of the care dependent people and their families. For care at home, long-term care insurance provides financial support if those affected choose to be cared for by relatives, friends or volunteers instead of an outpatient care service (1). People who are not entitled to benefits from long-term care insurance but are dependent on help and care must organize this through informal caregivers and/or through self-financed professional services. Informal care thus includes both the provision of care services supported by long-term care insurance and care and/or support in everyday life without the involvement of long-term care insurance. The long-term care situation might have positive or negative impact on the health situation of caregivers. Other studies found that caregivers are exposed to greater strains in their daily lives, which may affect physical and mental health and can be associated with increased stress and social isolation (2–5).

The aim of this study is to describe the social characteristics, the health and living situation and the prevalence of behavioral risk factors of adult informal caregivers compared to non-caregivers in Germany.

## 2. Materials and methods

### 2.1. Study design and sampling

The Robert Koch Institute regularly carries out surveys to monitor the health of the population in Germany. We used data from the GEDA 2019/2020-EHIS survey, which is a cross-sectional population-based health interview survey that was conducted between April 2019 and September 2020 using computer-assisted, fully-structured telephone interviews. The study population comprised people aged 15 or above living in private households, whose usual residence at the time of data collection was Germany. This includes both one- and multi-person households that operate independently and provide for their own needs. As such, collective households such as hospitals, care and residential homes, prisons, military barracks, religious institutions, boarding houses or hostels are not included in the survey. The survey used a telephone sample, which was provided by the Arbeitskreis Deutscher Markt- und Sozialforschungsinstitute e. V. (ADM). It is based on the so-called dual-frame method, in which two selection populations are used: one consisting of mobile phone numbers, and another consisting of landline phone numbers. This sampling method provides (almost) complete coverage of the population in Germany. A method developed by Leslie Kish for the random selection of respondents in multi-person households (the Kish Selection Grid,) was used to randomly select prospective respondents. Here, all potential interview partners are given the same selection probability and one person is randomly selected by the computer. This person is identified on the basis of the recorded age and gender. A total of 23,001 individuals with complete interviews participated in GEDA 2019/2020-EHIS (12,101 women, 10,838 men, 62 reported another gender identity or did not provide information). The response rate according to the standards of the American Association for Public Opinion Research was 21.6% (6). A detailed description of the methodology as well as of the classification of the response rate of GEDA 2019/2020-EHIS is available elsewhere (7). For our analyses we used data from all respondents with a female or male gender identity aged 18 years an older (*n* = 22,646).

### 2.2. Data protection and ethics

GEDA 2019/2020-EHIS is subject to strict compliance with the data protection provisions set out in the EU General Data Protection Regulation (GDPR) and the Federal Data Protection Act (BDSG). The Ethics Committee of the Charité – Universitätsmedizin Berlin assessed the ethics of the study and approved the implementation of the study (application number EA2/070/19). Participation in the study was voluntary. The participants were informed about the aims and contents of the study and about data protection. Informed consent was obtained verbally.

### 2.3. Measures

Internationally established instruments of the European Health Interview Survey (EHIS) were used to assess self-reported information on the provision of informal care or assistance, health status, behavioral risk factors, social support and sociodemographic characteristics (8).

#### 2.3.1. Provision of informal care

Respondents were asked, if they provide care or assistance to one or more persons suffering from some age problem, chronic health condition or infirmity, at least once a week. If they provided care, one further question assessed, for how many hours per week these respondents usually provide care or assistance (< 10 h per week; at least 10 but < 20 h per week; 20 h per week or more). We differentiated between providing no informal care (i.e., non-caregivers), informal care < 10 h/week (less-intense caregivers) and informal care at least 10 h/week (intense caregivers).

#### 2.3.2. Health status

The three questions of the Minimum European Health Module (MEHM) (9) include the *self-perceived health* by a single question ‘How is your health in general?' (very good, good, fair, bad, very bad), the presence of *chronic diseases* or a long-standing health problem lasting for 6 months or more (yes, no), and the *health-related activity limitations*. The latter was assessed using the Global Activity Limitation Indicator (GALI) *via* the question ‘Are you limited because of a health problem in activities people usually do?' (severely limited, limited, but not severely, not limited at all). Participants with limitations were additionally asked 'Have you been limited for at least the past 6 months?' (yes, no). Participants who had been limited for more than 6 months were defined as having longer-term health limitations. All other participants were considered to have no long-term limitations. The prevalence of a *low back disorder or other chronic back defect* in the past 12 months were assessed by a single question (yes, no). *Depressive symptoms* within the last 2 weeks were defined according to the German version of the 8-item Patient Health Questionnaire (PHQ-8; cut-off ≥10/24) (10).

#### 2.3.3. Behavioral risk factors

Individuals with alcohol consumption within the past 12 months were asked by a quantity-frequency measure separately for the amount of standard drinks consumed on weekdays (Mondays to Thursdays) and during weekends (Fridays to Sundays). The responses were used to calculate grams of pure alcohol consumed per day. *At-risk drinking* according to national guidelines (11, 12) was considered when >10/20 g pure alcohol per day was reported by women/men. Lower amounts were considered as low-risk alcohol consumption (including abstainers past 12 months or lifetime). Smoking status was assessed by a single question “Do you smoke tobacco products, including heated tobacco products?” *Current smoking* was defined for answers “yes, daily” or “yes, occasionally”. All other answer options (i.e., no, not any more, I have never smoked) were defined as current non-smoking. Work-related, transport-related and leisure-time physical activity in a typical week was assessed by the German version of the European Health Interview Survey – Physical Activity Questionnaire (EHIS-PAQ) (13). Respondents were asked about the duration of the physical activity they undertake during a typical week, in the form of both moderate-intensity aerobic physical activity conducted during leisure time and cycling used for transportation, as well as the number of days a week during which they undertake muscle-strengthening activities. *Insufficient physical activity* was defined as not meeting the recommendations of the World Health Organization on 2.5 hours of aerobic activity a week, as well as muscle-strengthening activities twice a week. Information on *non-daily fruit and vegetable consumption* was combined from two frequency questions regarding fruit and vegetable/salad consumption. A non-daily fruit and vegetables consumption was considered for those reporting a non-daily consumption of fruits or vegetables. *Obesity* (yes, no) was defined as a body mass index of ≥30 kg/m^2^ based on self-report of body weight and height according to the classification of the World Health Organization (14).

#### 2.3.4. Social characteristics

*Social support* was assessed using the OSLO-3 Scale (15) and categorized as low, moderate and high. Household size was dichotomized as living in a *single household* (yes, no). Participants were asked to indicate which *gender* they felt they belonged to (female, male, other gender identity) (16). Due to the small number of cases, participants who indicated a different gender identity or no gender identity were not included in the analyses. *Age* in years was categorized into two different groupings: (a) 18–44, 45–64 and >65 years and (b) 18–29, 30–44, 45–64, 65–79 and >80 years. *Educational levels* were assigned to low, medium, and high education groups according to the Comparative Analyses of Social Mobility in Industrial Nations (CASMIN) classification using school and vocational educational attainment (17, 18). *Municipality size* was categorized as rural (population < 5,000), small town (population 5,000 to < 20,000), medium town (population 20,000 to < 100,000), and city (population 100,000 and more) (reference date: 31 December 2018). *Current employment status* was differentiated into full-time and part-time employment, retirement and other (e.g., unemployed, being a student/pupil, fulfilling domestic asks, military or civilian service).

### 2.4. Data analysis

Weighted prevalences are presented overall or separately for women and men stratified by the provision of informal care or assistance with 95% confidence intervals (95% CI). Separate multinomial regression analyses were applied to determine group differences for caregivers and non-caregivers on health status, behavioral risk factors and social risk factors. In detail, intense and less-intense caregivers were compared with non-caregivers as the reference group. Regression analyses were calculated and adjusted for age group. Odds ratios are presented and significant *p*-values indicated.

The analyses were performed applying a weighting factor in order to correct for deviations of the sample from the population structure. As part of the data weighting, a design weighting was first performed for the different selection probabilities (mobile and landline network). Subsequently, an adjustment was made to the official population figures related to age, sex, federal state and type of district (reference date: 31 December 2019). In addition, the sample was adjusted to the education distribution in the 2017 Microcensus according to the International Standard Classification of Education (ISCED classification) (19).

All analyses were conducted using Stata 17.0 (Stata Corp., College Station, TX, USA, 2017). In order to take the weighting appropriately into account when calculating confidence intervals and *p*-values, all analyses were calculated using the survey procedures of Stata 17.0. A difference between groups was assumed to be statistically significant if the corresponding *p* < 0.05.

## 3. Results

### 3.1. Sample characteristics

In total, 51.1% were female, 38.8% were 18–44 years old, 52.4% had a medium education level, 33.8% lived in a city, 40.2% worked full-time (Table 1).

**Table 1:** Sample characteristics (total sample: *n* = 22,646).

	**N (% unweighted)**	**% weighted**	**95% CI**
**Gender**			
Female	11,959 (52.8)	51.1	50.1–52.1
Male	10,687 (47.2)	48.9	47.9–49.9
**Age groups in years**			
18–44	5,847 (25.8)	38.8	37.8–39.9
45–64	8,963 (39.6)	35.1	34.2–36.0
> 65	7,836 (34.6)	26.0	25.2–26.9
**Education level**			
Low	4,261 (18.8)	29.5	28.6–30.5
Medium	9,947 (43.9)	52.4	51.4–53.4
High	8,378 (37.0)	18.0	17.5–18.6
Missing	60 (0.3)		
**Municipality size**			
Rural	1,766 (7.8)	10.7	10.0–11.3
Small town	5,031 (22.2)	25.3	24.4–26.2
Medium town	5,805 (25.6)	30.2	29.3–31.2
City	8,503 (37.5)	33.8	32.9–34.7
Missing	1,541 (6.8)		
**Current employment status**			
Full–time employment	8,601 (38.0)	40.2	39.2–41.2
Part–time employment	3,564 (15.7)	15.6	14.9–16.3
Retirement	7,967 (35.2)	27.5	26.7–28.4
Other^a^	2,467 (10.9)	16.7	15.9–17.5
Missing	47 (0.2)		
**Informal Care**			
Intense caregivers (≥10 h/week)	1,573 (6.9)	6.5	77.5–79.1
Less-intense caregivers (< 10 h/week)	3,843 (17.0)	15.2	14.5–15.9
Non-caregivers	17,183 (75.9)	78.3	6.0–7.0
Missing	47 (0.2)		

^a^Unemployed, being a student/pupil, fulfilling domestic tasks, military or civilian service; 95% CI = 95% confidence interval; Percentages may not total 100 due to rounding.

### 3.2. Provision of informal care

Overall, 21.7% (*n* = 5,416) of the participants provided informal care or support for one or more persons suffering from age-related complaints, chronic illnesses or frailty at least once a week (Table 1). A total of 6.5% provided informal care at least 10 h per week (intense caregivers); 15.2% < 10 h per week (less-intense caregivers).

**Figure 1:**
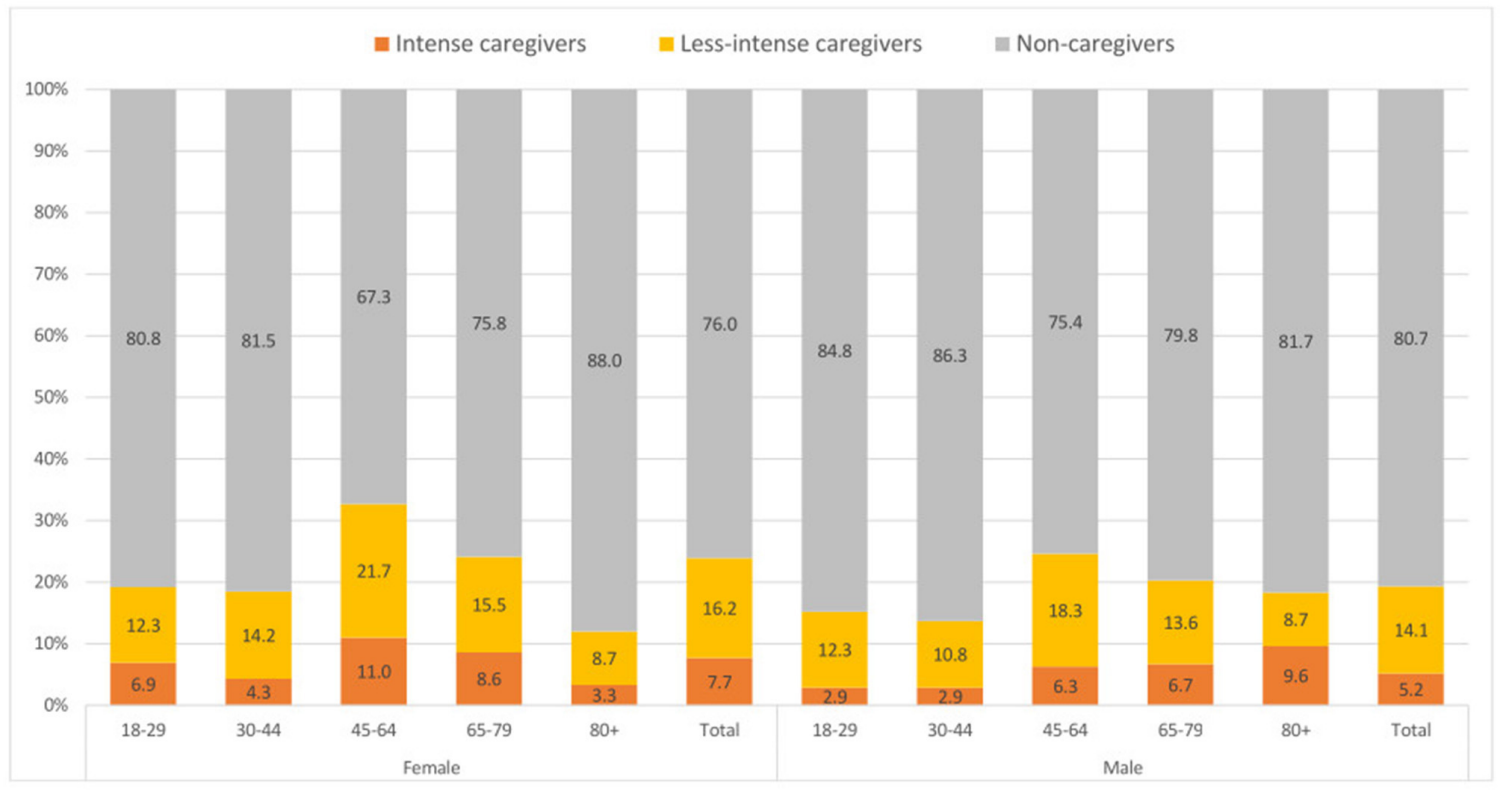
Percentage of informal care-giving activities by gender and age group in years (weighted analyses). Percentages may not total 100 due to rounding.

Women provided informal care more often (23.9%) than men (19.3%) (Table 2). Informal caregiving is most frequently provided in the age group of 45 to 64 years, among both women and men: 32.7% of women and 24.6% of men of that age stated that they supported or cared for others (Figure 1).

**Table 2:** Social characteristics by provision of informal care (weighted analyses).

		**Intense caregivers**		**Less–intense caregivers**		**Non–caregivers**	
		**%**	**95% CI**	**%**	**95% CI**	**%**	**95% CI**
**Female**	**Total**	7.7	7.1–8.5	16.2	15.3–17.2	76	74.9–77.1
	**Age groups in years**						
	18–44	25.5	20.9–30.7	30.6	27.4–34.0	39.4	37.8–41.1
	45–64	49	44.3–53.8	46	42.9–49.1	30.5	29.1–31.9
	> 65	25.5	21.8–29.5	23.4	21.1–25.9	30.1	28.7–31.5
	**Education level**						
	Low	34.2	29.5–39.2	20.8	18.2–23.8	29.4	27.8–31.0
	Medium	55.9	51.0–60.6	63.7	60.7–66.6	53.9	52.3–55.5
	High	10	8.4–11.7	15.4	13.9–17.1	16.7	15.9–17.6
	**Municipality size**						
	Rural	14.3	11.1–18.2	11.5	9.6–13.9	9.9	8.9–10.9
	Small town	28.4	24.0–33.2	25.8	23.1–28.7	25	23.6–26.5
	Medium town	29.1	24.9–33.6	33.2	30.1–36.4	30.3	28.8–31.9
	City	28.3	24.1–32.9	29.5	26.7–32.5	34.8	33.3–36.3
	**Current employment status**						
	Full–time job	23.4	19.7–27.7	31.6	28.7–34.7	27.1	25.7–28.6
	Part–time job	28.8	24.6–33.4	29.6	26.8–32.5	23.1	21.9–24.4
	Retirement	26.9	23.1–31.1	23.9	21.6–26.5	31.5	30.1–33.0
	Other^a^	20.8	17.0–25.3	14.9	12.5–17.6	18.2	16.9–19.6
**Male**	**Total**	5.2	4.6–5.9	14.1	13.1–15.1	80.7	79.6–81.8
	**Age groups in years**						
	18–44	22.9	17.8–28.8	33.2	29.4–37.2	43.3	41.7–45.0
	45–64	43.8	37.6–50.1	46.7	42.9–50.5	33.5	32.0–35.1
	> 65	33.4	27.8–39.5	20.2	17.6–23.0	23.1	21.9–24.4
	**Education level**						
	Low	41.1	34.7–47.7	29.6	25.9–33.6	30.2	28.6–31.9
	Medium	45.6	39.4–52.0	51	47.2–54.8	49	47.3–50.6
	High	13.3	10.9–16.1	19.4	17.5–21.6	20.8	19.9–21.8
	**Municipality size**						
	Rural	7.5	4.9–11.3	12	9.6–14.9	10.9	9.8–12.0
	Small town	36.8	30.4–43.7	24.9	21.7–28.3	24.5	23.1–26.0
	Medium town	27.8	22.5–33.8	31	27.4–34.8	29.6	28.1–31.2
	City	27.9	22.6–33.9	32.2	28.7–35.9	34.9	33.4–36.5
	**Current employment status**						
	Full–time employment	40.4	34.4–46.8	56.6	52.9–60.4	53.8	52.2–55.4
	Part–time employment	6.3	3.9–10.0	7.9	5.9–10.5	5.9	5.2–6.7
	Retirement	38.6	32.6–44.9	23	20.3–25.9	24.4	23.1–25.7
	Other^a^	14.7	10.6–20.1	12.5	9.8–15.7	15.9	14.6–17.3

^a^Unemployed, being a student/pupil, fulfilling domestic tasks, military or civilian service; 95% CI = 95% confidence interval; Percentages may not total 100 due to rounding.

### 3.3. Health status and the provision of informal care

The analyses reveal that female intense caregivers were significantly more likely to have a low back disorder or other chronic back defects than female non-caregivers (43.0 vs. 32.4%). There were no significant differences between these two groups and all other variables concerning health status (Table 3).

**Table 3:** Health status of caregivers and non–caregivers by gender (weighted analyses).

		**Intense caregivers**		**Less-intense caregivers**		**Non-caregivers**		**Intense care vs. no care (Ref.)**	**Less intense care vs. no care (Ref.)**
		**%**	**95% CI**	**%**	**95% CI**	**%**	**95% CI**	**OR** ^a^	**OR** ^a^
**Female**	**Self–perceived health**								
	Very good/good	63.1	58.2–67.6	71.4	68.3–74.2	68.6	67.1–70.1	Ref.	Ref.
	Fair/bad/very bad	36.9	32.4–41.8	28.6	25.8–31.7	31.4	29.9–32.9	1.19	0.84^*^
	**Health-related activity limitations**								
	No	59.6	54.8–64.1	67	64.0–70.0	64.4	62.8–65.9	Ref.	Ref.
	Yes	40.4	35.9–45.2	33	30.0–36.0	35.6	34.1–37.2	1.12	0.84^*^
	**Chronic disease**								
	No	42.9	38.2–47.7	47.4	44.2–50.6	48.6	47.1–50.2	Ref.	Ref.
	Yes	57.1	52.3–61.8	52.6	49.4–55.8	51.4	49.8–52.9	1.14	0.99
	**Low back disorder or other chronic back defect**								
	No	57	52.1–61.7	63.4	60.2–66.4	67.6	66.1–69.0	Ref.	Ref.
	Yes	43	38.3–47.9	36.6	33.6–39.8	32.4	31.0–33.9	1.47^**^	1.16
	**Depressive symptoms**								
	No	89.2	85.6–92.0	91.3	88.8–93.3	91.4	90.3–92.3	Ref.	Ref.
	Yes	10.8	8.0–14.4	8.7	6.7–11.2	8.6	7.7–9.7	1.25	0.98
**Male**	**Self–perceived health**								
	Very good/good	56	49.5–62.3	74.7	71.2–77.9	72	70.4–73.5	Ref.	Ref.
	Fair/bad/very bad	44	37.7–50.5	25.3	22.1–28.8	28	26.5–29.6	1.61^**^	0.79^*^
	**Health-related activity limitations**								
	No	52.3	45.9–58.6	69.1	65.5–72.6	70	68.4–71.5	Ref.	Ref.
	Yes	47.7	41.4–54.1	30.9	27.4–34.5	30	28.5–31.6	1.71^***^	0.94
	**Chronic disease**								
	No	41.2	35.2–47.6	53.5	49.7–57.2	54.5	52.8–56.1	Ref.	Ref.
	Yes	58.8	52.4–64.8	46.5	42.8–50.3	45.5	43.9–47.2	1.39^*^	0.96
	**Low back disorder or other chronic back defect**								
	No	58.2	51.9–64.4	66.9	63.2–70.4	71.3	69.8–72.8	Ref.	Ref.
	Yes	41.8	35.6–48.1	33.1	29.6–36.8	28.7	27.2–30.2	1.57^**^	1.16
	**Depressive symptoms**								
	No	91.6	86.7–94.9	91.8	88.8–94.1	92.6	91.5–93.6	Ref.	Ref.
	Yes	8.4	5.1–13.3	8.2	5.9–11.2	7.4	6.4–8.5	1.15	1.06

^a^Separate multinomial logistic regression analyses adjusted for age group; 95% CI = 95% confidence interval; Ref., reference group; OR, odds ratios; ^*^p < 0.05; ^**^p < 0.01; ^***^p < 0.001.

Male intense caregivers reported also significantly more often a low back disorder or other chronic back defect (41.8 vs. 28.7%) compared to male non-caregivers. In addition, they indicated more often fair/bad/very bad self-perceived health (44.0 vs. 28.0%), health-related activity limitations (47.7 vs. 30.0%), and the presence of chronic diseases (58.8 vs. 45.5%) than male non-caregivers. No significant differences were found concerning depressive symptoms (Table 3).

Female and male less-intense caregivers showed significantly less often a fair/bad/very bad self-perceived health compared to female and male non-caregivers (females: 28.6 vs. 31.4%; males: 25.3 vs. 28.0%). Among women, it was also found that less-intense caregivers had significantly fewer health-related activity limitations than non-caregivers (33.0 vs. 35.6%). No significant differences were found for the other variables concerning health status (Table 3).

### 3.4. Behavioral risk factors and the provision of informal care

There were no significant differences between female and male intense caregivers compared to female and male non-caregivers concerning behavioral risk factors (Table 4).

**Table 4:** Health behavioral risk factors of caregivers and non–caregivers by gender (weighted analyses).

		**Intense caregivers**		**Less-intense caregivers**		**Non-caregivers**		**Intense care vs. no care (Ref.)**	**Less intense care vs. no care (Ref.)**
		**%**	**95% CI**	**%**	**95% CI**	**%**	**95% CI**	**OR^a^**	**OR^a^**
**Female**	**At-risk drinking**								
	No	89.6	86.3–92.2	89	87.2–90.6	88.8	87.8–89.7	Ref.	Ref.
	Yes	10.4	7.8–13.7	11	9.4–12.8	11.2	10.3–12.2	0.89	0.95
	**Current smoking**								
	No	72.2	67.4–76.5	75	71.9–78.0	76.5	75.1–78.0	Ref.	Ref.
	Yes	27.8	23.5–32.6	25	22.0–28.1	23.5	22.0–24.9	1.23	1.03
	**Insufficient physical activity**								
	No	19.4	16.1–23.2	27.9	25.1–30.8	22.8	21.5–24.1	Ref.	Ref.
	Yes	80.6	76.8–83.9	72.1	69.2–74.9	77.2	75.9–78.5	1.22	0.77^**^
	**Non-daily fruit and vegetable consumption**								
	No	48.1	43.4–52.9	48	44.9–51.2	44.1	42.5–45.7	Ref.	Ref.
	Yes	51.9	47.1–56.6	52	48.8–55.1	55.9	54.3–57.5	0.84	0.84^*^
	**Obesity**								
	No	76.3	71.8–80.3	81.5	78.9–83.9	81.4	80.0–82.6	Ref.	Ref.
	Yes	23.7	19.7–28.2	18.5	16.1–21.1	18.6	17.4–20.0	1.24	0.93
**Male**	**At-risk drinking**								
	No	86.8	81.8–90.5	83.9	80.9–86.5	83.7	82.4–84.9	Ref.	Ref.
	Yes	13.2	9.5–18.2	16.1	13.5–19.1	16.3	15.1–17.6	0.74	0.96
	**Current smoking**								
	No	64.8	58.3–70.8	65.9	61.9–69.6	66.2	64.6–67.9	Ref.	Ref.
	Yes	35.2	29.2–41.7	34.1	30.4–38.1	33.8	32.1–35.4	1.28	1.02
	**Insufficient physical activity**								
	No	23.3	18.7–28.5	35.5	31.9–39.4	28.7	27.2–30.1	Ref.	Ref.
	Yes	76.7	71.5–81.3	64.5	60.6–68.1	71.3	69.9–72.8	1.1	0.65^***^
	**Non-daily fruit and vegetable consumption**								
	No	26.6	21.5–32.4	26.8	23.7–30.2	23.4	22.1–24.7	Ref.	Ref.
	Yes	73.4	67.6–78.5	73.2	69.8–76.3	76.6	75.3–77.9	0.86	0.82^*^
	**Obesity**								
	No	75.8	69.8–81.0	79.2	75.8–82.3	81.5	80.2–82.8	Ref.	Ref.
	Yes	24.2	19.0–30.2	20.8	17.7–24.2	18.5	17.2–19.8	1.29	1.08

^a^Separate multinomial logistic regression analyses adjusted for age group; 95% CI = 95% confidence interval; Ref., reference group; OR, odds ratios; ^*^p < 0.05; ^**^p < 0.01; ^***^p < 0.001.

Less-intense caregivers showed a more favorable health behavior than non-caregivers. They were significantly less often physically inactive (females: 72.1 vs. 77.2%; males: 64.5 vs. 71.3%) and their fruit and vegetable consumption was significantly less likely to be non-daily (females: 52.0 vs. 55.9%; males: 73.1 vs. 76.6%). There were no significant differences concerning at-risk drinking, current smoking, and obesity (Table 4).

### 3.5. Social risk factors and the provision of informal care

Both female and male caregivers (regardless of the extent of care provided) lived significantly less often alone compared to non-caregivers (females: intense caregivers 23.6%, less-intense caregivers 32.5%, non-caregivers 40.7%; males: intense caregivers 33.3%, less-intense caregivers 37.8% non-caregivers 43.2%) (Table 5).

**Table 5:** Social risk factors of caregivers and non–caregivers by gender (weighted analyses).

		**Intense caregivers**		**Less-intense caregivers**		**Non-caregivers**		**Intense care vs. no care (Ref.)**	**Less intense care vs. no care (Ref.)**
		**%**	**95% CI**	**%**	**95% CI**	**%**	**95% CI**	**OR^a^**	**OR^a^**
**Female**	**Single household**								
	No	76.4	71.6–80.6	67.5	64.3–70.6	59.3	57.7–60.9	Ref.	Ref.
	Yes	23.6	19.4–28.4	32.5	29.4–35.7	40.7	39.1–42.3	0.42^***^	0.72^***^
	**Social support**								
	Moderate/high	88.1	84.6–90.8	89.8	87.2–92.0	84.7	83.3–85.9	Ref.	Ref.
	Low	11.9	9.2–15.4	10.2	8.0–12.8	15.3	14.1–16.7	0.75	0.63^**^
**Male**	**Single household**								
	No	66.7	59.7–73.1	62.2	58.1–66.2	56.8	55.1–58.5	Ref.	Ref.
	Yes	33.3	26.9–40.3	37.8	33.8–41.9	43.2	41.5–44.9	0.80^*^	0.65^**^
	**Social support**								
	Medium/high	79.2	71.9–85.0	88.2	84.9–90.8	83.3	81.8.−84.6	Ref.	Ref.
	Low	20.8	15.0–28.1	11.8	9.2–15.1	16.7	15.4–18.2	1.23	0.65^**^

^a^Separate multinomial logistic regression analyses adjusted for age group; 95% CI = 95% confidence interval; Ref., reference group; OR, odds ratios; ^*^p < 0.05; ^**^p < 0.01; ^***^p < 0.001.

Low social support was significantly less common among female and male less-intense caregivers compared to non-caregivers (females: 10.2 vs. 15.3%, males: 11.8 vs. 16.7%). No significant differences were found between intense caregivers and non-caregivers concerning social support (Table 5).

## 4. Discussion

About one fifth of the respondents provide informal care, mainly at least 10 h per week. Women provide informal care more often than men. Caregivers most often belong to the age group 45 to 64 years. Intense caregivers more often suffer from back pain than those who provide less or no care. Men who provide intense care are more likely to report fair/bad/very bad self-perceived health status, health related limitations in daily living and chronic diseases than non-caregivers. Female and male less-intense caregivers were less often physically inactive and non-daily fruits and vegetables consumption was less likely compared to non-caregivers. The majority of caregivers do not live alone. Low social support is not as common among less-intense caregivers as among non-caregivers.

It must be considered that there is no international consensus on how the indicator of informal long-term care should be implemented in survey studies. A comparison of the studies EHIS, The Survey of Health, Aging and Retirement in Europe (SHARE) and the European Quality of Life Survey (EQLS) shows a very inconsistent picture. Based on different question wordings there are remarkable differences in the level of informal long-term care provision in the population and the differences between countries hardly follow a clear pattern (20). The EHIS definition used here is very broad and includes not only long-term care activities in the narrower sense but also other, not further defined support services in daily life. In the present analyses, it was assumed that frequent provision of support (≥10 h per week) suggests a regular activity with daily or almost daily caregiving and therefore comes closer to the construct of informal care. Nevertheless, it must be stated that a clear definition of informal long-term care is still missing especially on the European level (21). The present study allows to describe the group of informal caregivers in more detail with regard to the extent of care provided and their social characteristics, health status, and possible risk and protective factors, and to compare them with the group of non-caregivers.

There are only a few cross-sectional studies on the social characteristics, health and living situation and the prevalence of behavioral risk factors of adult informal caregivers in Germany. The proportion of informal caregivers that was identified in these studies (22, 23) is similar to the present results. Consistent with our findings, the existing studies also show that women provide informal care more often than men and that the proportion of caregiving increases with age (22–24). This finding is also confirmed by international study results (25).

A current systematic review suggests that informal caregiving may be associated with adverse health related outcomes like several mental and physical disorders, including pain (26). This is line with our results for men: With the exception of depressive symptoms, intense caregivers are more likely to report worse health outcomes than non-caregivers. For women, we found significant differences in health status only for back pain, which is consistent with the research findings (27). The fact that we did not find more adverse health outcomes for caregiving women compared to non-caregivers should be further investigated. Apparently, female caregivers and female non-caregivers differ less in different health status characteristics than male caregivers and male non-caregivers do. Focusing future research on differences within gender groups could provide new insights into this. In summary, it should be emphasized that the main burden of care work is to be found in middle age and that possible health-promoting and relieving measures should not least focus on this group. Furthermore, noticeable gender differences should be considered and investigated further. In the present study, the negative effects of care work on the health of caring men are striking. Thus, future research should also clarify the extent to which gender-related approaches to health promotion and prevention could be promising for informal carers.

Regarding behavioral risk factors, our results show hardly any differences between intensive caregivers respectively less-intense caregivers, and non-caregivers. The exceptions are insufficient physical activity and non-daily fruit and vegetable consumption where differences are found between less intensive caregivers and non-caregivers in favor of the less-intense caregivers. That caregiving is associated with health-promoting behaviors is supported by previous findings (28). However, in contrast to our results, these findings indicate increased risk behaviors among caregivers, e.g. related to obesity and smoking. Our findings that non-daily consumption of fruits and vegetables and insufficient physical activity are less common among less-intensive caregivers compared with intensive caregivers or non-caregivers are also confirmed by others (29). A recent systematic review (30) aimed at better understanding of physical activity of caregivers. The authors conclude that the current body of research is insufficient to assess whether informal caregivers are at higher risk for physical inactivity than non-caregivers. They recommend further research with validated measures for the different domains of physical activity (leisure time, daily physical activity, caregiving duties). And it should be noted that we only consider healthy diet on the basis of one indicator, which does not adequately reflect the complexity of nutrition.

Overall, with the exception of back pain the results do not suggest consistent major negative effects of caregiving on health status for women and men. Similarly, intense caregivers did not report having worse health-related lifestyles than non-caregivers. Less-intense caregivers report even better health than non-caregivers. One explanation to understanding these associations is that healthier people are more likely to take on caregiving tasks, while those with poorer health are less likely to do so (healthy caregiver effect) (31, 32). Further, received social support could also help avoid a burden (33). Otherwise, it cannot be excluded that the definition of informal care that was implemented in EHIS may not be sufficiently specific to clearly distinguish caregivers with a high care burden from those caregivers that frequently spend time with their relatives while being supported in care activities by professional services and thus have a much lower care burden. This could weaken the association between informal caregiving and health. Finally, caregivers who experience high levels of burden are probably less likely to participate in a health survey due to time constrains as an analysis of reasons for non-participation among individuals 65 years and older suggest (34). We therefore assume that the proportion of caregivers with health problems could be underestimated.

In addition, due to the demographic change and population aging we are expecting higher numbers of people in need of care (35). Researches from the European Joint Research Center estimate that the number of people aged 50 years and older with long-term care needs will increase by approximately 24% by 2050 and 36% by 2070 (36). The major part of care will continue to be provided by informal caregivers. A structured review showed that despite the important role of informal care, few studies have included this aspect of care into their demand models (37). Therefore, their health status and burden should be regularly monitored in order to develop prevention strategies to avoid negative health effects.

### 4.1. Strengths and limitations

The results refer to a large nation-wide population-based sample of 22,646 respondents aged 18 years and older. Possible factors associated with selection bias have been considered by weighting according to age, sex and education (7). Nevertheless, the following limitations have to be considered. The first wave of the 2020 COVID-19 pandemic was coincident within the survey period of this study. It cannot be completely ruled out that a change in willingness to participate during the pandemic has had an impact on certain health indicators. The present analyses were done under the assumption that the sample does not show systematic bias due to the containment measures. Moreover, initial analyses do not show a systematic selection between the subsamples of the comparison periods 2019 and 2020. We therefore suggest that the data collection during the pandemic did not represent an exceptional period with significant impact on the level of care-relevant indicators (38).

However, it must be considered that GEDA 2019/2020-EHIS was not primarily aimed at informal carers. For example, we lack detailed information on whom and why somebody is cared for and also former caregiving activities. Therefore, we cannot give more insight in (a) the reported gender differences of intense caregivers regarding their relationship with the person cared for; i.e., support of partners vs. non-partners or (b) the care needs and strains of care. Furthermore, healthy people may be more likely to provide informal care and that they may stop doing so when their health deteriorates.

Another related limitation of the study is that we cannot distinguish between respondents with friends or family members in need of care who actively provide care and those who don't but delegate this to third parties like professional care services. The willingness to provide informal care can vary due to many factors such as degree of kinship, career orientation, time constraints, distance between one's own residence and that of the person to be cared for. This alone may entail a selection between informal caregivers and non-caregivers, which should be taken more into account in future studies.

### 4.2. Conclusion

Our study results show that in Germany a significant proportion of people provide informal care. Even though the present study did not show any serious health effects on those providing informal care, it can be assumed that they experience burden, especially when care is provided over a longer period of time. Preventive measures are important and should be supported in any way in order to maintain physical and mental health of informal care-givers. With the expected increase in the number of people needing care, protecting those who provide care is an important part of meeting future challenges.

## Data availability statement

The dataset presented in this article is not readily available because the authors confirm that some access restrictions apply to the data underlying the findings. The data set cannot be made publicly available because informed consent from study participants did not cover public deposition of data. However, the minimal data set underlying the findings is archived in the “Health Monitoring” Research Data Centre at the Robert Koch Institute (RKI) can be accessed by researchers on reasonable request. On-site access to the data set is possible at the Secure Data Center of the RKI's “Health Monitoring” Research Data Centre. Requests should be submitted to the “Health Monitoring” Research Data Centre, Robert Koch Institute, Berlin, Germany (e-mail: fdz@rki.de).

## Ethics statement

The study involving human participants was reviewed and approved by the Ethics Committee of the Charité – Universitätsmedizin Berlin (application number EA2/070/19). Informed consent was obtained verbally. Written informed consent for participation was not required for this study in accordance with the national legislation and the institutional requirements.

## Author contributions

All authors listed have made a substantial, direct, and intellectual contribution to the work and approved it for publication.
